# Co-immobilization of Cellulase and β-Glucosidase into Mesoporous Silica Nanoparticles for the Hydrolysis of Cellulose Extracted from *Eriobotrya japonica* Leaves

**DOI:** 10.1021/acs.langmuir.2c00053

**Published:** 2022-04-27

**Authors:** Giulio Pota, Antonio Sapienza Salerno, Aniello Costantini, Brigida Silvestri, Jessica Passaro, Valeria Califano

**Affiliations:** † Department of Chemical, Materials and Production Engineering, University of Naples Federico II, Piazzale Tecchio 80, 80125 Fuorigrotta, Naples, Italy; ‡ Institute of Science and Technology for Sustainable Energy and Mobility (STEMS), National Research Council of Italy (CNR), Viale Marconi 4, 80125 Naples, Italy

## Abstract

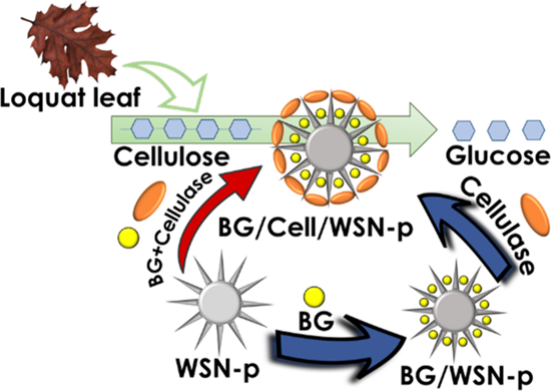

Fungal cellulases generally contain a reduced amount of β-glucosidase (BG), which does not allow for efficient cellulose hydrolysis. To address this issue, we implemented an easy co-immobilization procedure of β-glucosidase and cellulase by adsorption on wrinkled mesoporous silica nanoparticles with radial and hierarchical open pore structures, exhibiting smaller (WSN) and larger (WSN-p) inter-wrinkle distances. The immobilization was carried out separately on different vectors (WSN for BG and WSN-p for cellulase), simultaneously on the same vector (WSN-p), and sequentially on the same vector (WSN-p) in order to optimize the synergy between cellulase and BG. The obtained results pointed out that the best biocatalyst is that prepared through simultaneous immobilization of BG and cellulase on the same vector (WSN-p). In this case, the adsorption resulted in 20% yield of immobilization, corresponding to an enzyme loading of 100 mg/g of support. 82% yield of reaction and 72 μmol/min·g activity were obtained, evaluated for the hydrolysis of cellulose extracted from *Eriobotrya japonica* leaves. All reactions were carried out at a standard temperature of 50 °C. The biocatalyst retained 83% of the initial yield of reaction after 9 cycles of reuse. Moreover, it had better stability than the free enzyme mixture in a wide range of temperatures, preserving 72% of the initial yield of reaction up to 90 °C.

## 1 Introduction

Lignocellulosic agricultural waste biomass is considered a strategic fuel source. In this frame, hydrolysis catalyzed by cellulase enzymes to produce glucose represents a powerful tool.

Second-generation bioethanol fuel can be obtained by fermentation of glucose derived from lignocellulosic biomass. The use of ethanol instead of gasoline as transportation fuel provides a more complete and cleaner combustion, reducing carbon monoxide, unburned hydrocarbon, and particulate emissions.^1,2^ Furthermore, bio-derived ethanol used as fuel can help mitigate climate change, being CO_2_ neutral.^3^

Lignocellulosic biomass is the most abundant non-edible source of glucose due to its high content of cellulose. It is readily available from industry and agricultural waste with no ethical concern of food competition.^4^ Contrary to conventional sources of glucose, i.e., starch, sugar cane, and sugar beet, the whole plant can be used to obtain sugars.^5^ However, cellulose is much more difficult to hydrolyze, due to the β-bonds that join the glucose units. This type of bond forces the cellulose macromolecules into a linear conformation. The cellulose chains then join in microfibrils held together by hydrogen bonds, which makes cellulose recalcitrant to enzymatic hydrolysis.^6^ In fact, substances such as starch, which contain glucose polymers with α-bonds, serve as an energy reserve and glucose is readily available. Cellulose, on the other hand, is a structural polymer, which constitutes the cell wall of plants, and is resistant to biological attack. Furthermore, the cellulose in the biomass is embedded in hemicellulose and lignin in a composite structure. Lignin is a complex phenolic polymer that provides a physical barrier for enzymatic hydrolysis and adsorbs cellulolytic enzymes on its sticky surface.^7,8^ For these reasons, the enzymatic hydrolysis of cellulose normally requires a pre-treatment to make it more available to cellulolytic enzymes.^9^ The pre-treatment process should efficiently remove lignin and reduce the cellulose degree of polymerization and crystallinity, as amorphous cellulose is more readily hydrolyzed by enzymes.^10^

Enzymatic hydrolysis of biomass is generally preferred over chemical methods since it is a green route avoiding toxic reagents and byproducts and facilitates downstream processing. The enzymes responsible for the hydrolysis of cellulose are called cellulase. Cellulase, a multi-enzyme system, consists of three types of enzymes, which act synergistically in the decomposition of cellulose. Endo-glucanase (EG) and cellobiohydrolase (CBH) act on insoluble cellulose producing soluble oligomers, i.e., cellobiose and cellotriose. β-Glucosidase (BG) hydrolyzes the β-bond of soluble oligosaccharides, leading to the formation of glucose. However, the use of cellulase in soluble form has various drawbacks, mainly related to its intrinsic instability and the difficulty of multiple reuses. In addition, continuous operation is not possible. These are essential issues to offset what is one of the major problems of enzymatic catalysis: the high cost of enzymes.^11^ One useful strategy to overcome these limitations is the immobilization of the enzyme on an insoluble support. This allows exploiting the advantages of heterogeneous catalysis, such as the possibility of reuse and working in continuous operation, but it also has additional benefits. In fact, with a properly designed immobilization protocol, immobilized enzymes can gain stability with respect to harsh environmental conditions, such as high temperatures and pH far from neutral.^12,13^ There are several reasons for enzyme stabilization upon immobilization, i.e., the rigidification of the enzyme structure following multipoint attachment or for interaction with the pore walls in porous structures; the seclusion of the enzyme from the external environment by immobilization in porous materials, which protects the enzyme from denaturing factors; or an actual conformational variation due to the interaction with the support, which leads to greater stability.^14^ Furthermore, immobilization in some cases can cause an increase in the activity and specificity of the enzyme.^13,15^ The increase in the activity of an enzyme as a result of immobilization occurs more rarely. Sometimes it is caused by structural alterations of the enzyme that accidentally increase its activity in a certain reaction; other times, it is simply the result of the greater stability of the enzyme, so that the activity is higher when measured under drastic conditions. Finally, immobilized enzymes are dispersed on the support surface, which prevents aggregation encountered with free enzymes resulting in a decline in activity.^15^

However, immobilization of cellulase is challenging because cellulose is water-insoluble. To carry out its catalytic role, cellulase must diffuse to the cellulose surface. Immobilized cellulase has very low mobility, so that diffusion limitations become an issue.^16^ For this reason, immobilization of cellulase is often performed with nonporous materials, which have the advantage that the enzyme molecules are on the surface of the carrier and can thus have access to insoluble cellulose.^17^ Enzyme orientation is another critical point since only when the active center is properly oriented to the medium, the enzyme can perform its catalytic action.^18^ Several solutions have been proposed to enhance cellulase flexibility so to favor the right orientation, such as the use of a long spacer arm,^19^ immobilization on flexible polymer brush^20,21^ temperature-responsive polymers,^22^ or biomimetic anemone-inspired supports.^23^ Another problem that must be considered when using cellulase for biomass conversion is that often BG activity is scarce in the enzymatic cocktail.^24^ BG relieves the inhibition exerted by cellobiose on cellulolytic enzymes; hence, its role is essential for efficient hydrolysis of the biomass. All the three enzymes carry out their action interdependently, so that the enzyme composition in the enzymatic cocktail must be well balanced. Co-immobilization of exogenous BG and cellulase is a possible solution. In fact, it was shown that supplementing commercial cellulases with BG increased the yield of glucose.^25^ One of the first attempts at co-immobilization of the two systems was done by one-pot entrapment/covalent immobilization in a polyurethane foam.^26^ Although the co-immobilized enzymes performed better than cellulase immobilized alone, the reaction yields were far from those obtained with free enzymes. In another study, β-glucosidase and cellulase were simultaneously and covalently co-immobilized on a pH-responsive copolymer.^27^ The immobilized enzymes showed better glucose yield (62.69% after 72 h) compared with the free enzymes in the hydrolysis of microcrystalline cellulose. More recently, sequential co-immobilization of BG and cellulase has been performed on hierarchical microparticles and layered films.^21,28^ BG was entrapped in the inner core of a poly(ethylene glycol) layer, and cellulase was covalently bound on the outer surface of a brush polymer layer to improve the accessibility of insoluble cellulose to the enzyme and preserve cellulase flexibility.

A wide plethora of materials can be used as a support for enzyme immobilization. The choice should be guided by different features, such as cost, availability, stability, porosity, surface area, and above all the affinity between the enzyme and the carrier.^29^ The support for enzyme immobilization can be inorganic (i.e., silica or titania), synthetic organic (mostly polymers), or organic of natural origin.^30−32^ Mesoporous silica nanoparticles stand as the adequate supports for enzyme immobilization thanks to their large surface area, narrow pore size distribution, well-defined pore geometry, and thermal and mechanical stability. Moreover, they exhibit water insolubility, renewability, and toxicological safety. The main technique to produce them is the sol–gel route: the mild synthesis conditions allow preparing sophisticated hybrid organic–inorganic systems where the synergic characteristics and functionalities of a single component extend and improve the properties of the final material.^33,34^ Their numerous hydroxyl groups can be activated allowing for enzyme covalent attachment.^35,36^ SBA-15 has been the first mesostructured silica material used to immobilize cellulase enzymes^37^ thanks to its pore, large enough to host bulky enzymes. FDU-12 materials are particularly well-suited for enzyme immobilization due to very large pores and high pore connectivity. Hartono et al. synthesized a series of organo-functionalized FDU-12 with very large pores up to 28 nm for the immobilization of cellulase by physical adsorption.^38^ The best biocatalyst showed high activity (70% of the free enzyme activity in the hydrolysis of carboxymethyl cellulose (CMC)). Chang et al. immobilized cellulase from *Trichoderma reesei* by both physical adsorption and covalent binding on synthesized ultralarge pore LP (20–40 nm) silica nanoparticles.^39^ The biocatalytic assay was carried out on cellulose oligomers, obtained through a pre-treatment with the ionic liquid method.^40^ They found that the glucose yields reached by the covalently immobilized biocatalyst were 83.79%, against a glucose yield of approximately 85% provided by free cellulase. Moreover, it showed high storage stability, giving a glucose yield of 86.56% after 23 days storage at room temperature. In our previous work,^41^ we immobilized cellulase by adsorption on wrinkled silica nanoparticles (WSNs). WSNs were synthesized by using pentanol as a co-solvent (WSN-p), in order to enhance the inter-wrinkle distance and properly host the enzyme. The prepared biocatalyst was assayed in the hydrolysis of CMC, providing the same activity as the free enzyme. In this work, BG and cellulase were co-immobilized by a simple adsorption procedure on WSN-p to promote the hydrolysis of cellulose extracted from *Eriobotrya japonica* leaves**.** The choice of the support was guided by two considerations: (i) BG immobilized in WSNs showed enhanced activity^42,43^ and (ii) cellulase immobilized in WSN-p showed the same conversion rate of free cellulase and good operational stability.^41^

Despite the undoubted kinetic advantages that there can be in co-immobilizing two or more enzymes that perform simultaneous and synergistic action, this choice is not always the most appropriate compared to immobilizing these enzymes on different supports. This is because different enzymes can have different stability, size, and optimal reaction and immobilization conditions.^12,17^ Furthermore, the loading capacity of the support with respect to each enzyme is more limited.^17^ However, cellulase and BG have similar stability and optimal reaction conditions.^41,42^ The real problem here consists in obtaining a catalyst in which EG and CBH can perform their action on a bulky and insoluble substrate, cellulose. In the present paper, this challenge was faced by developing various and simple immobilization strategies using porous hierarchical supports to immobilize the two enzymes. The morphology of these supports allowed cellulase to easily attack a large and insoluble substrate such as cellulose and facilitated the diffusion of the soluble substrate (cellobiose) in the inner pores where BG could act. The immobilization was carried out (i) separately on different vectors (WSN for BG and WSN-p for cellulase) by adsorption of each enzyme on its support in separate batches, (ii) simultaneously on the same vector (WSN-p), by adsorption of the two enzymes on the same support in the same batch of adsorption, and (iii) sequentially on the same vector (WSN-p) by adsorbing first BG and then cellulose in a multilayer immobilization. The order for the layered immobilization was chosen to obtain the immobilization of BG in the inner core of the pores and cellulase toward the large pore entry, facilitating cellulase attack.

## 2 Experimental

### 2.1 Materials

Tetraethylorthosilicate (TEOS), urea, cetyltrimethylammonium bromide (CTAB), cyclohexane, pentanol, 2-propanol, ethanol, hydrochloric acid solution (37.0% wt in water), carboxymethylcellulose sodium salt (CMC), acetic acid (99.0% wt), sodium acetate trihydrate, sodium hydroxide and glucose oxidase-peroxidase (GOD-POD) assay kit, citric acid, trisodium citrate dihydrate, and sulfuric acid (95.0–98.0% wt) were purchased from Sigma-Aldrich (Milan, Italy). β-Glucosidase from almonds (molecular weight of 135 kDa for the dimer, product number 49290, specific activity of ≥4 U/mg, measured as micromole of glucose liberated per minute at pH 5 and 37 °C with salicin as substrate) and cellulase from *T. reesei* (product number C0615, specific activity of ≥5 U/mg solid measured as micromole of glucose liberated from cellulose per hour at 37 °C and pH 5) were also acquired from Sigma-Aldrich. Sodium hypochlorite solution (5.0% wt) was bought from a local supermarket. *E. japonica* (loquat) leaves were collected from a private garden in Caserta, Italy.

### 2.2 Cellulose Extraction

Cellulose was extracted from *E. japonica* leaves following a two-step procedure.^44^ Dry leaves were collected from the ground and kept in a ventilated oven at 40 °C for 24 h to remove moisture. 2 g of the dried biomass were cut into smaller pieces, put into a dry cloth, grounded to fine pellets, and finally dispersed in 60 mL of sodium hydroxide water solution (4% wt). The system was kept under stirring at 80 °C for 2 h. Afterward, the suspension was centrifuged and washed three times with bidistilled water. This first step was repeated thrice. The second step aimed at bleaching purified cellulose using a bleaching solution made of equal volumes of distilled water, acetic acid/sodium acetate trihydrate buffer (pH = 5), and sodium hypochlorite 1.7% wt. The solid fraction coming from step one was dispersed into 60 mL of the bleaching solution. The system was kept under stirring at 80 °C for 2 h. The samples were collected by centrifugation and washed three times with distilled water. This routine was repeated 4 times. Finally, bleached cellulose was dried in a ventilated oven at 40 °C for 24 h. The final product of the extraction was a crispy and fragile white film.

### 2.3 WSN Synthesis

WSNs and WSN-p were synthesized following the procedure described by Moon and Lee^45^ using CTAB instead of cetylpyridinium bromide (CPB) as the surfactant. The peculiar morphology of WSN-p was achieved by replacing 2-propanol with pentanol as the co-solvent. Briefly, cyclohexane (oil phase) and a co-solvent (2-propanol and pentanol for WSN and WSN-p, respectively) were added to a water solution of urea and CTAB (surfactant) under stirring. The reaction mixture evolved into a Winsor III system, characterized by a bicontinuous microemulsion phase stabilized by CTAB. Afterward, TEOS was added dropwise and the hydrolysis/condensation series of reactions started at the microemulsion interface. The system was kept under stirring at 70 °C for 24 h. Subsequently, a surfactant-removal step was carried out dispersing the nanoparticles in a mixture of HCl and ethanol at 70 °C for 24 h. The result was the formation of a hierarchical mesoporous silica architecture, with a central-radial porous structure. The final product was collected by centrifugation and washed three times with ethanol. Quantitative information of the preparation procedure is reported in our previous works.^41,46^

### 2.4 Optimization of BG/Cellulase Ratio

The BG/cellulase weight ratio was optimized using free cellulase and BG as biocatalysts and CMC as the substrate for the hydrolysis reaction. Supplementary BG was needed to enhance the glucose production by pushing forward the conversion of the cellobiose produced as the reaction intermediate. First, four different enzyme mixtures were tested in the hydrolysis of CMC (concentration set to 2 mg/mL), in order to identify the optimal weight ratio between the enzymes. The composition of each enzyme mixture is reported in Table 1.

**Table 1:** Composition of the Enzyme Mixtures Used for the Hydrolysis of 2 mg/mL CMC

enzyme mixture	BG (mg/mL)	cellulase (mg/mL)
A	0	2.0
B	0.40	2.0
C	0.67	2.0
D	1.0	2.0

Hydrolysis reactions were carried out in citric acid/sodium citrate buffer (pH = 5, 50 mM) at 50 °C, under mild stirring. In detail, 5 mL of enzyme mixture (A, B, C, and D alternatively) was added to 5 mL of a 20 mg/mL CMC buffer solution. The reaction mixture was withdrawn from the reactor after 24 h, thermally inactivated in an oven at 100 °C for 10 min, and then submitted to spectrophotometric analysis for the determination of glucose concentration. The percentage increment of obtained glucose (Δ glucose) was calculated as follows:
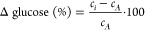
where *c*_i_ and *c*_A_ are the concentration of glucose produced by using biocatalyst enzyme mixture i (i = B, C, D, alternatively) and enzyme mixture A (which is absolute cellulase).

### 2.5 Enzyme Immobilization

BG and cellulase were physically immobilized onto two different matrices: WSN and WSN-p. Physical immobilization was carried out in citric acid/sodium citrate buffer (pH = 5, 50 mM). In both cases, a 4 mg/mL buffer suspension of the support was prepared and mixed with an equal volume of a 2 mg/mL enzyme buffer solution. The system was kept under mild stirring at 40 °C for 24 h. Temperature and time of immobilization were optimized in a previous study.^41^ The supported biocatalysts (BG/WSN and cellulase/WSN-p) were collected by centrifugation, washed twice with bidistilled water, and stored as wet pellets at 4 °C.

One-pot co-immobilization was carried out in the same conditions but using a solution containing both enzymes. The support chosen was WSN-p since it was shown that the secondary structure of cellulase was better preserved in WSN-p than in WSN.^41^ Briefly, a solution of BG and cellulase was prepared by dissolving both enzymes in the same citrate buffer. The enzyme concentration was set to 0.33 mg/mL for BG and 2 mg/mL for cellulase. The aim was to design a supported biocatalyst with the same enzyme composition as enzyme mixture C (Table 1). Afterward, 16.5 mL of the enzyme solution was added to an equal volume of a 4 mg/mL suspension of WSN-p in buffer and the system was kept under mild stirring at 40 °C for 24 h. Finally, the biocatalyst was collected and washed as usual. The concentration ratio between BG and cellulase in the enzyme solution was fixed to 1:6 since we initially assumed that the yield of immobilization of each enzyme, when co-immobilized on WSN-p, remained the same as the one achieved when the proteins are immobilized separately, each on the corresponding support (30% for BG/WSN and 15% for cellulase/WSN-p).^41,42^

Sequential co-immobilization was accomplished by splitting the immobilization process of the two enzymes into two consecutive steps. In the first one, a 0.66 mg/mL BG buffer solution was added to an equal volume of a 4 mg/mL WSN-p buffer suspension. The resulting biocatalyst (SEQ-BG) was collected by centrifugation, washed twice with bidistilled water, and dispersed in citrate buffer to a final support concentration of 4 mg/mL. Afterward, an equal volume of a 2 mg/mL cellulase buffer solution was added to the system. The final biocatalyst was collected, washed, and stored as described above. The supported biocatalysts were referred to as SEP-BG/cell, SIM-BG/cell, and SEQ-BG/cell, depending on whether they were obtained through separate immobilization, simultaneous (one pot), or sequential co-immobilization, respectively.

The effectiveness of the adsorption for each sample was determined by thermogravimetric analysis (TGA) by subtracting the organic content of each support from the one of the corresponding immobilized biocatalysts. The yield of immobilization (YI %) was calculated as the weight ratio between the adsorbed enzyme and the amount dissolved in the adsorption mixture, in percentage. TGA measurements were repeated after reuse cycles.

### 2.6 Pre-treated Biomass Hydrolysis

Both free and supported enzymes were employed in the hydrolysis of pre-treated biomass. The reactions were carried out under mild stirring at 40 °C and pH 5. All the catalytic assays were carried out with cellulase concentration set to 1 mg/mL. For free enzymes, BG concentration was alternatively set to 0.33 and 0.2 mg/mL for BG:cellulase wt/wt equal to 0.33 and 0.2, respectively. The amount of supported biocatalyst used in the reaction was similarly selected in order to have cellulase concentration equal to 1 mg/mL. As a consequence, the BG concentration was equal to 0.2 mg/mL for SEQ-BG/cell and included in the 0.2–0.33 mg/mL range for SIM-BG/cell. In detail, 10 mg of pre-treated biomass was cut into small pieces and dispersed into 5 mL of each free enzyme mixture. The system was allowed to react for 24 h, kept in a circulating oven (100 °C, 10 min) to thermally deactivate the protein, and analyzed to determine the obtained glucose concentration. The free enzyme-catalyzed hydrolysis reaction was also carried out on the untreated loquat leaf for comparison. Operating conditions (*T*, time) and cellulase concentration was set the same as above. Briefly, 58 mg of dry loquat leaves was ground into fine pieces and added to 5 mL of BG/cellulase free enzyme mixture, with BG:cellulase w/w equal to 1:5. The amount of untreated biomass was chosen in order to have cellulose concentration equal to 2 mg/mL, being loquat leaf chemical composition reported in the literature.^47^ The reaction was stopped after 24 h, and the glucose concentration was estimated as previously reported.

Cellulose hydrolysis was carried out with separately immobilized enzymes on two different vectors (SEP-BG/cell), one-pot (SIM-BG/cell) and sequential (SEQ-BG/cell) co-immobilized enzymes on the same vector. The reaction conditions were the same as the free enzymes but the reaction mixture was centrifuged to separate the biocatalyst before analyzing the glucose concentration. The amount of each supported biocatalyst was chosen in order to reproduce the same composition as the free enzyme mixture. One-pot (SIM-BG/cell) and sequentially (SEQ-BG/cell) co-immobilized biocatalysts were similarly tested. Results were expressed in terms of yield of reaction (YR %), calculated as the concentration ratio between glucose and the organic component of the substrate, in percentage. The specific activity of both free and supported enzymes was evaluated toward cellulose and expressed as μmol/min of obtained glucose per gram of enzyme. The amount of glucose was measured after 30 min since it was the minimum time for cellulose to be significantly dissolved by enzyme aggression, as confirmed by visual detection. Activity measurements were carried out in the same reaction conditions chosen to evaluate long-time glucose production. Enzyme concentration and operating conditions were almost overlapped to those set for adsorption, thus satisfying the basic requirements for a successful immobilization.^48^

### 2.7 Operational and Thermal Stability

The operational stability was assessed by submitting the supported biocatalyst to 24 h consecutive reaction cycles on pre-treated biomass at 50 °C and pH 5. The results were expressed in terms of relative glucose production (%) with the glucose concentration after the first reaction cycle chosen as the reference. After each reaction cycle, the biocatalyst was collected by centrifugation and washed once with bidistilled water.

Thermal stability evaluation was accomplished by incubating the supported biocatalyst for 1 h at a given temperature (60, 70, 80, 90, and 100 °C) before reacting with cellulose for 24 h at 50 °C. The yield of reaction obtained without any incubation phase was chosen as the reference to evaluate the residual yield of reaction (%) after incubation at temperature *x*.

### 2.8 Experimental Techniques

The evolution of the morphology experienced by the nanosystems during the immobilization steps was investigated through transmission electron microscopy (TEM), using a FEI Tecnai G2 20 Microscope (FEI, Hillsboro,OR, USA).

The enzyme loading of the nanoparticles was assessed by thermogravimetric analysis (TGA). Approximately 10 mg of dried samples was ground, loaded into a platinum pan, and submitted to a temperature ramp from 30 to 1000 °C under an air atmosphere, with a heating rate of 10 °C/min. The organic weight fraction (O %) of each sample was evaluated as follows:
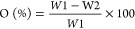
1where *W*1 and *W*2 refer to the sample weight at 30 and 1000 °C, respectively. The experiments were performed in a TA Instrument Q600SDT apparatus. TGA was also used to estimate the organic weight fraction of the pre-treated biomass. 5 mg of the samples was settled on the bottom of a platinum pan and submitted to the same temperature ramp as the supported biocatalysts under an air atmosphere. The percentage contribution of organic compounds within the biomass was calculated following equation 1. The residual weight is attributable to the ash fraction.

N_2_ adsoprtion/desorption experiments were performed on WSN-p before and after BG and BG/cellulase adsorption. Experiments were carried out at −196 °C with a Quantachrome autosorb iQ, after degassing for 4 h at 80 °C. The specific surface area of the samples was calculated by the Brunauer–Emmett–Teller (BET) method.

The effectiveness of the cellulose extraction process was assessed by Fourier-transform infrared spectroscopy (FTIR) in the attenuated total reflection (ATR) mode, using a Nexus FTIR spectrometer provided with a DuraSam-plIR II accessory equipped with a ZnSe crystal. The spectra of pristine and pre-treated loquat leaves were recorded in the range 4000–525 cm^–1^ at a spectral resolution of 4 cm^–1^. The spectrum of Whatman filter paper was acquired for comparison.

FTIR allowed also for detecting the presence of enzyme molecules into the silica nanostructure after each step of sequential immobilization. The spectrometer was equipped with a DTGS (deuterated triglycine sulfate) KBr detector. Pristine WSN-p, SEQ-BG, and SEQ-BG/cell dry powder were ground, pressed into pellets (13 nm in diameter), and submitted to spectral recording (4000–400 cm^–1^ wavenumber range, 2 cm^–1^ spectral resolution, 32 scans for each acquisition). The blank KBr spectrum was acquired as the background.

A glucose (GO) assay kit was used to estimate the concentration of glucose obtained from the reaction. The experimental procedure is the d-glucose oxidase–peroxidase method.^49^ Aliquots of the reaction product were withdrawn from the reactor and diluted 1:10 with bidistilled water. 300 μL of each diluted solution was poured in an Eppendorf tube, mixed to 600 μL of glucose-measuring reagent, and kept in a thermostatically controlled water bath at 37 °C for 30 min. Finally, 600 μL of sulfuric acid (12 N) was added to the system before measuring the absorbance at 540 nm using a SHIMADZU UV-2600i spectrometer. A calibration curve was built in order to calculate the glucose concentration values from the absorbance measurements.

## 3 Results and Discussion

### 3.1 Biomass Pre-treatment

The recalcitrance of cellulose to biological attack is due both to the presence of lignin and to the compactness of the cellulose fibers, which hinder enzyme penetration.^7^ For this reason, a pre-treatment of the biomass is necessary before enzymatic hydrolysis, aimed at eliminating lignin and reducing the degree of crystallinity of the cellulose. In fact, cellulose exists in four polymorphs.^50^ Of these, cellulose I occurs in nature and is made up of rather compact cellulose fibrils, intercalated by amorphous regions. Cellulose II can be obtained by alkaline treatment of cellulose I.^51^ Cellulose II is less crystalline than cellulose I, which favors enzymatic hydrolysis.^6^

Alkali pre-treatment of the biomass generally offers several advantages over other pre-treatment procedures, such as acid or biological pre-treatments.^6^ It requires milder conditions and is more environmentally friendly with respect to acid pre-treatments, which can also produce toxic substances for hydrolytic enzymes,^52^ and it can efficiently remove lignin within a few hours compared to greener biological pre-treatments that can take many days.^53^ However, its efficacy depends on the lignin content of the biomass: high lignin content will not be removed effectively.^54^ The treatment has in fact proven effective for fibers of the herbaceous plant *Syngonanthus nitens*, with a low content of lignin (6.5%),^44^ but not for sugar palm fibers with a lignin content of 13.4%.^55^ Lignin content of loquat leaves is estimated to be 19.2% of the overall lignocellulosic fraction.^47^ A consequent bleaching step was considered necessary to enhance cellulose weight fraction in the pre-treated biomass, in order to make it more available for enzyme aggression.

**Figure 1:**
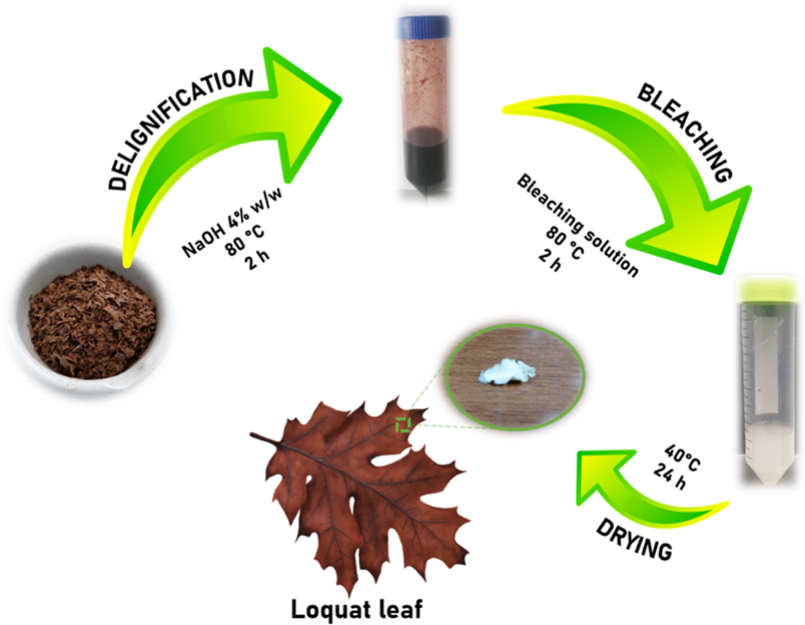
Graphical sketch of the biomass pre-treatment procedure.

Figure 1 shows pictures of the various steps used in the pre-treatment of loquat leaves.

After the first repetition of the delignification stage, the biomass suspension became wine-colored due to the release of lignin and other polyphenols pigment. Delignification is achieved through the saponification of ester bonds between lignin and hemicellulose.^56^ The bleaching treatment is expected to complete or at least push forward the cellulose purification by eliminating all the coloring compounds untouched by the delignification stage and the remaining lignin. In detail, the decomposition of sodium hypochlorite produces chlorine dioxide (ClO_2_), a strong oxidizer. The decomposition of sodium hypochlorite is favored at high temperature and in acidic pH. ClO_2_ oxidizes the aromatic rings of lignin producing lower–molecular-weight compounds, increasing its solubility. The color of the alkali-treated biomass turned white soon after the first repetition of the bleaching stage was completed. After the fourth repetition, the recovered bleached biomass appeared as a white pellet easily dispersible in water. This might be a consequence of the partial cellulose depolymerization caused by the oxidizing environment the biomass was submitted to.

**Figure 2:**
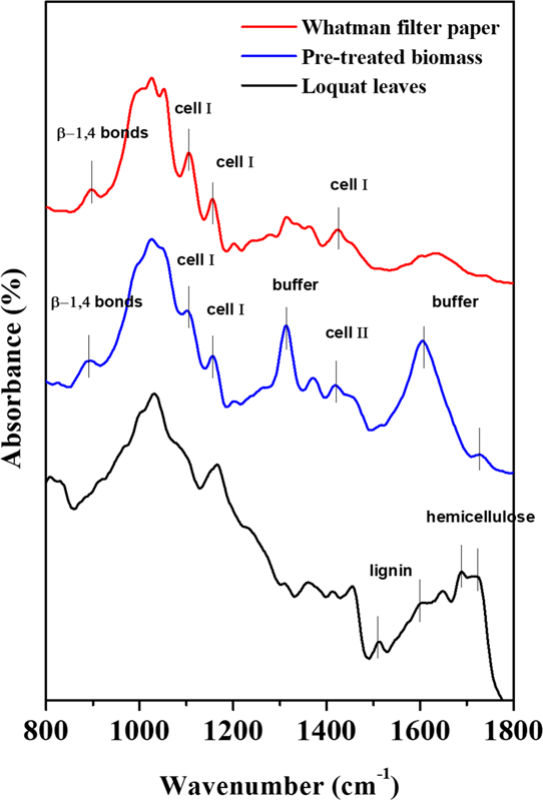
ATR spectra of loquat leaves (black curve), pre-treated biomass (blue curve), and Whatman filter paper (red curve) displayed in the 800–1800 cm^–1^ wavenumber range.

Figure 2 shows FTIR spectra of a loquat leaf, pre-treated biomass, and filter paper as a reference for type I crystalline cellulose.

The biomass spectrum has a double peak at 1687 and 1730 cm^–1^ due to the stretching vibration of the C=O bond of the acetyl groups of the hemicelluloses. The bands at 1603 cm^–1^ belong to the aromatic skeletal vibrations and the C=C stretching vibrations in lignin; aromatic skeletal vibration of lignin shows also an adsorption band at 1511 cm^-1.^^57^ The band at 1646 cm^–1^ is due to O–H bending vibration of adsorbed water. The rest of the spectrum are due to the overlapping of the bands of the different biomass components and are therefore difficult to interpret. Following the basic pre-treatment and bleaching, the biomass shows the characteristic cellulose fingerprint between 850 and 1500 cm^–1^, as can be seen by comparison with the spectrum of the filter paper. In particular, the peaks at 894 cm^–1^ represent −COC vibration at the β-glycosidic bond of cellulose,^58^ whereas the bands at 1105, 1156, and 1422 cm^–1^ are due to pyranose ring asymmetric stretching, C–O–C asymmetric stretching, and CH_2_ symmetric bending vibration of cellulose I.^59^ Cellulose I bands at 1105 and 1156 cm^–1^ are present in the pre-treated biomass spectrum, indicating that this crystalline form exists within the pre-treated biomass. However, the band at 1422 cm^–1^ is shifted at 1413 cm^–1^. This shift indicates that part of cellulose I is transformed into cellulose II and amorphous cellulose.^10^ The pre-treatment therefore produces a reduction in the degree of crystallinity of cellulose, making it more accessible to cellulolytic enzymes. The band at 1740 cm^–1^, present in the spectrum of the pre-treated biomass but not in that of the filter paper, indicates that part of the hemicellulose is still present after the pre-treatment.^10^ This fraction will probably not be fully converted by cellulase, as total biodegradation of xylan contained in hemicelluloses requires the action of different enzymes (endo-β-1,4-xylanase, β-xylosidase, and several accessory enzymes).^60^

The two intense peaks that stand out above the spectrum of the pre-treated biomass at 1312 and 1600 cm^–1^ obviously do not belong to any component of the biomass. In fact, neither lignin, nor cellulose, nor hemicellulose shows such intense peaks at those wavelength values. These bands could be associated with the presence of the trisodium acetate ions of the buffer loaded in the bleaching solution, which exhibits its most intense absorption at those wavelengths due to the symmetric and anti-symmetric stretching of COO^–^.^61^

### 3.2 Optimization of the BG/Cellulase Weight Ratio

Three families of enzymes that work synergistically to convert cellulose to glucose compose cellulase. CBH acts on the free ends of the cellulose chains, releasing mainly cellobiose, thus providing the substrate for BG that hydrolyzes it to glucose. EG is active on the amorphous regions of cellulose, randomly cutting internal linkages, creating new free ends for the action of CBH, and releasing soluble cellodextrins that will be hydrolyzed by BG. On the other end, the action of BG is essential since cellobiose can severely decrease the rate of cellulose hydrolysis, being an inhibitor of the cellulase complex.^62^ The synergy between the three enzymes is expressed on several levels.

**Figure 3:**
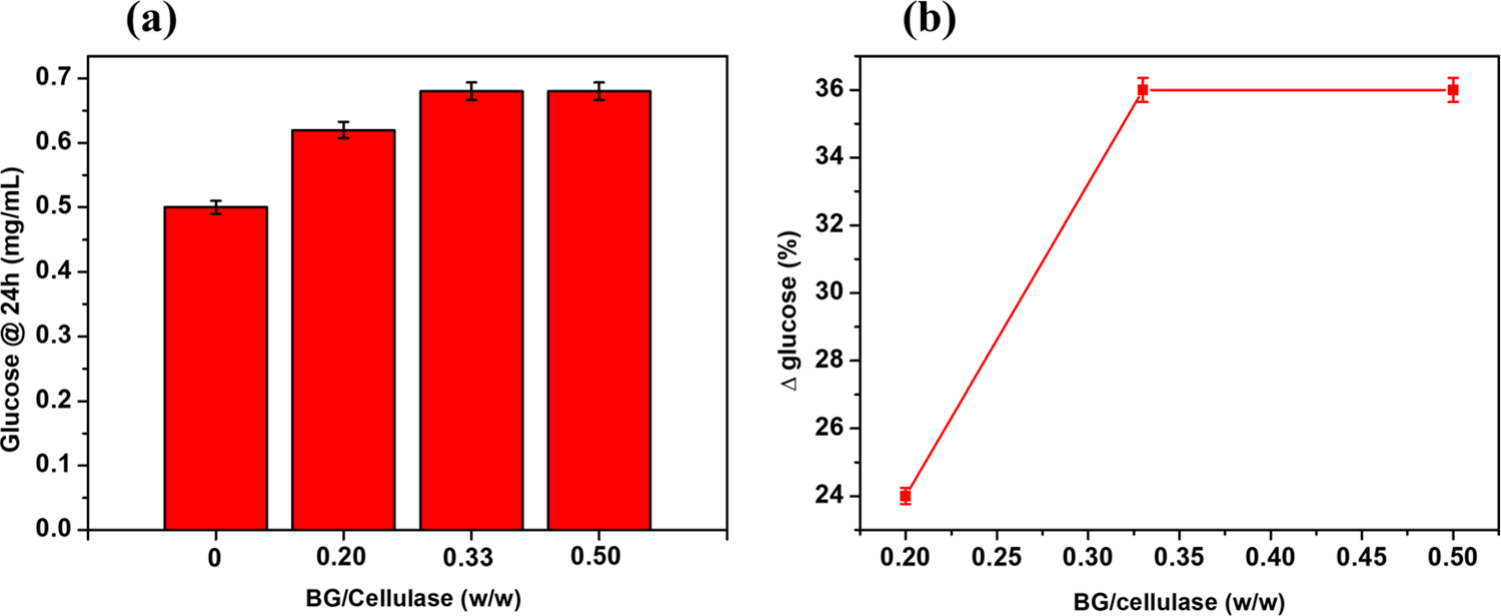
Histograms showing the concentration of glucose in the reaction mixture after 24 h obtained by enzyme mixtures of different compositions (a). Percentage increment of obtained glucose versus BG/cellulase w/w (glucose concentration produced by pure cellulase was set as reference) (b). Each experiment was performed in triplicate.

*T. reesei* cellulase, the most used fungal cellulase, contains 80% CBH and 12% EG.^63^ It is therefore clear that BG is insufficient for efficient hydrolysis and cellobiose will accumulate inhibiting the reaction.^24^ To obtain a high glucose yield, it is necessary to supplement the enzyme cocktail with additional BG. In the literature, there are several studies dealing with supplementation of free cellulase with free BG,^63,64^ immobilized BG,^65,66^ or co-immobilization of BG and cellulase^21,26−28^ using a BG/cellulase ratio of 0.1–0.5. In order to balance the enzyme cocktail improving the glucose yield, we carried out CMC hydrolysis varying the BG/cellulase ratio from 0.2 to 0.5. The results are presented in Figure 3a.

Histograms report the concentration of the glucose originating from the hydrolysis of CMC (2 mg/mL) in a reaction time of 24 h. Absolute cellulase is responsible for a glucose concentration of 0.50 mg/mL. Glucose concentration rises up to 0.62 mg/mL when the BG/cellulase weight ratio is set to 0.20, resulting in a 24% increase with respect to the performance of pure cellulase (Figure 3b). The percentage enhancement of the glucose production lowers to 9.7% when the weight ratio between the enzymes is pushed up to 0.33 with respect to the case of 0.20 w/w, resulting in a 36% overall increase. No further benefits are observed using a BG/cellulase w/w equal to 0.50, meaning that all the cellobiose produced as an intermediate is hydrolyzed to glucose (Figure 3b). Other authors have worked to optimize the enzyme cocktail composition before. For instance, Chakrabarti and Storey obtained a 3-fold higher glucose concentration by degrading CMC (1% w/v) with a mixture of BG (2 U) and cellulase (30 U) with respect to pure cellulase, in solution as well as co-immobilized into a polyurethane foam.^26^ Borges et al. proved that supplementing free cellulase (40 FPU/g_cellulose_) with immobilized BG (120 U/g _cellulose_) resulted in 40% higher conversion of sugarcane bagasse to glucose in 96 h.^66^ Moreover, supplementing free cellulase with BGs extracted from six different fungi (BG/cellulase = 0.4 w/w) was found to enhance filter paper conversion of 4.22 times.^63^ The enzyme cocktail composed of 0.50 U/mL BG and 0.75 U/mL cellulase was found to be effective in enhancing corn straw conversion to glucose by 94% with respect to absolute cellulase.^67^ Glucose production from the hydrolysis of microcrystalline cellulose was increased by 8.3% after supplementing commercial cellulolytic formulation (5 U/mL cellulase, 0.45 U/mL BG) with 0.40 U/mL of purified BG from *Candida peltata*.^68^ Based on literature results and on our experiments, a BG/cellulase ratio between 0.20 and 0.33 w/w can be enough to optimize the biomass hydrolysis yield with the enzymes in immobilized form.

### 3.3 Enzymes Co-immobilization

It is widely known that endoglucanase and exoglucanase have high affinity for cellulose surfaces, which make them easily recoverable by adsorption on fresh cellulose.^69^ On the contrary, BG does not adsorb on cellulose. BG should be made readily available where the endoglucanases and exoglucanases have performed their action, to avoid cellobiose accumulation in the proximity of the two enzymes with consequent inhibition. BG will also be inhibited by both cellobiose and its reaction product, glucose. However, at the chosen concentrations (the maximum glucose concentration obtained is approximately 9 mM), the inhibition is limited.^42^

**Figure 4:**
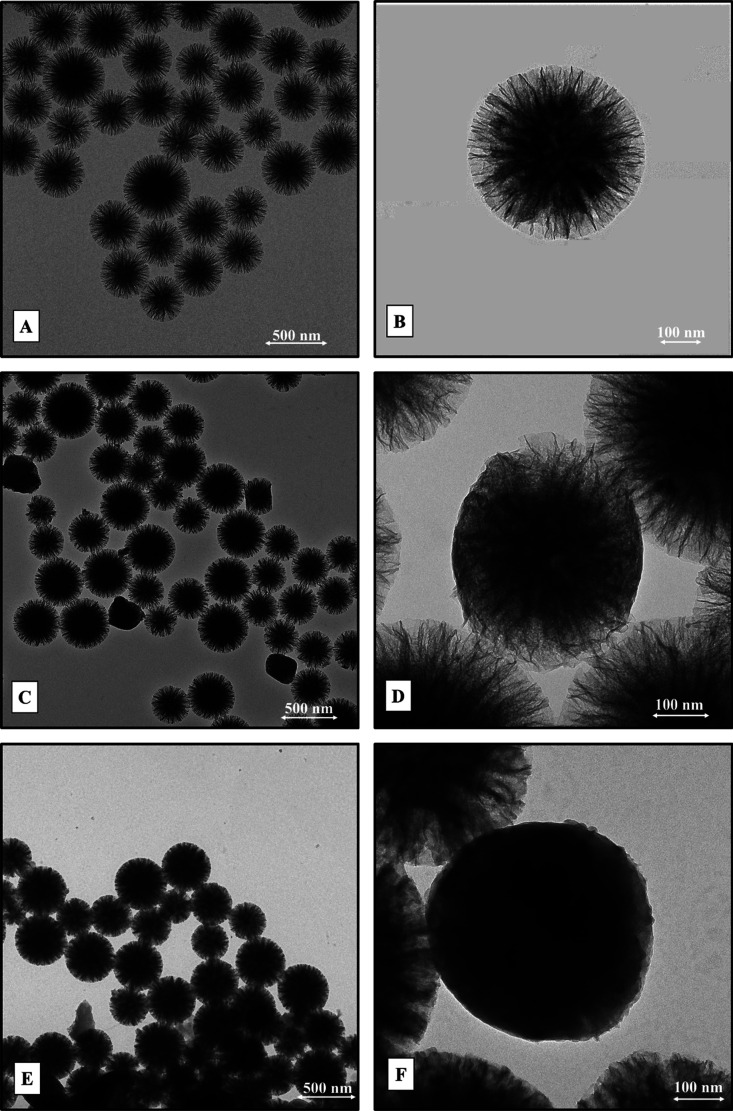
TEM images for WSN-p (A,B), SEQ-BG (C,D), and SEQ-BG/cell (E,F) taken at lower (500 nm, left column) and higher (100 nm, right column) magnifications.

Consequently, we used different co-immobilization strategies to enhance the synergistic action exerted by the enzymes in the hydrolysis reaction of cellulose. As determined by the BG/cellulase ratio optimization tests, we tried to obtain the immobilization in the ratio 0.33 w/w of the two enzymes. To determine the ratio actually obtained, the samples were subjected to thermogravimetric analysis. In particular, the SEQ-BG/cell sample was analyzed after the first adsorption stage (SEQ-BG) and in the second stage of cellulase adsorption on the BG-filled sample (SEQ-BG/cell). TEM and FTIR investigations were also carried out on bare WSN-p, SEQ-BG, and SEQ-BG/cell, in order to observe the presence of BG in the nanoparticles and the degree of filling thereof. Figure 4 reports TEM micrographs for all the nanosystems.

**Figure 5:**
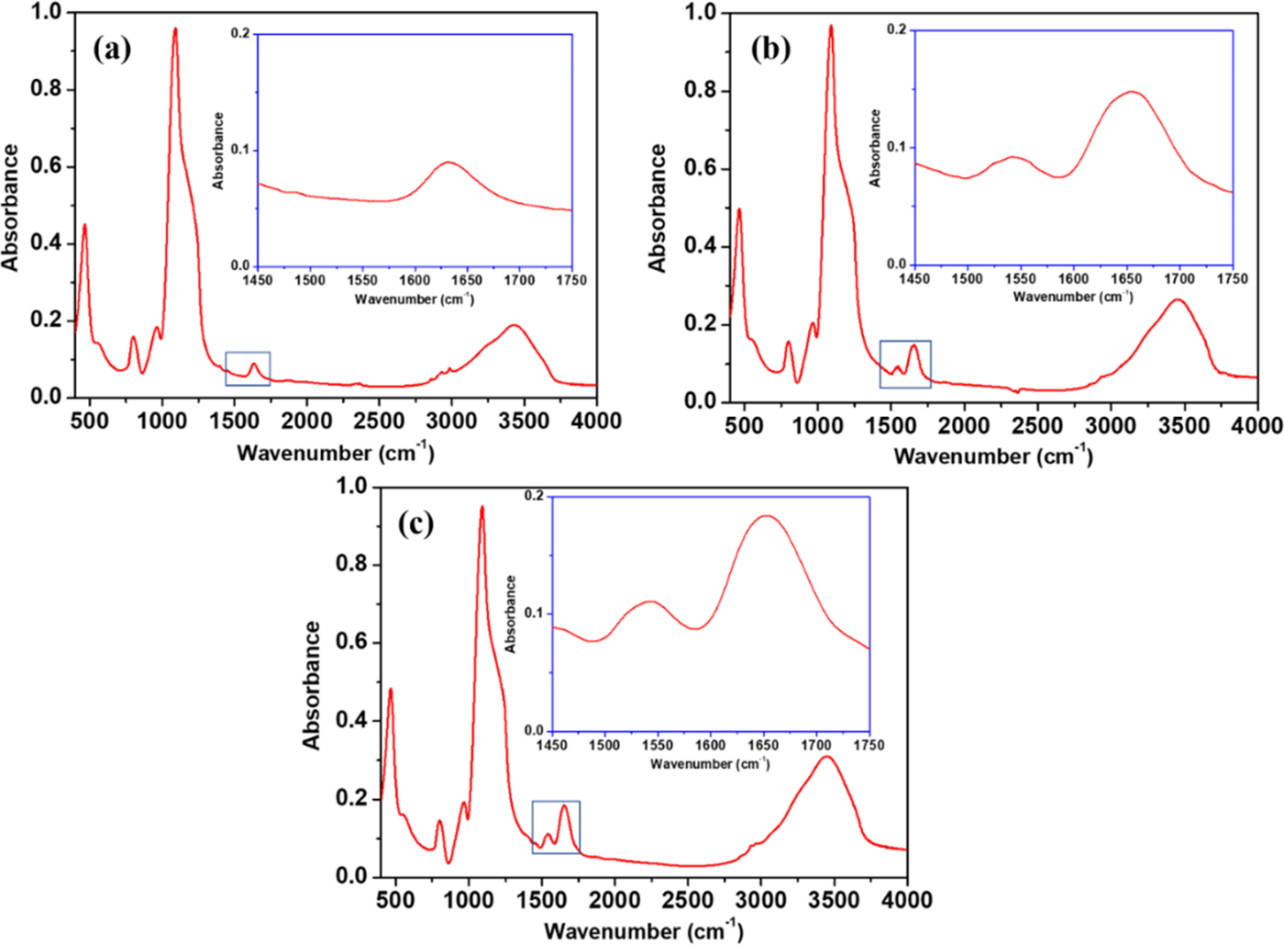
FTIR spectra for WSN-p (a), SEQ-BG (b), and SEQ-BG/cell (c). Insets in blue rectangles show the focus on the amide I/amide II region (1450–1750 cm^–1^).

WSN-p exhibited the well-known profile, showing radial silica nanofibers with enhanced inter-wrinkle distance if compared to WSNs, as already discussed in our previous study.^41^ The image taken at lower magnification (Figure 4A) shows that the nanoparticles appear as a rather monodisperse system with a diameter in the 300–500 nm size range. Figure 4C,D proves the presence of BG inside the mesopore structure of the silica support due to the increased contrast visible in the inner core of SEQ-BG nanosystems. In particular, BG homogeneously settles along the entire length of pores in WSN-p (Figure 4D) during the first immobilization step. Micrographs referring to the SEQ-BG/cell sample (Figure 4E,F) show that subsequent adsorption of cellulase totally fills the pore structure of the support, as highlighted by the net contrast increase experienced by the nanostructure surface: the immobilized protein almost completely hides the profile of the silica support (Figure 4F). However, the overall diameter of the nanostructure does not change after the immobilization process since no protein corona layer of appreciable thickness is formed over the support. The effectiveness of each adsorption stage is confirmed by FTIR spectra reported below in Figure 5.

WSN-p exhibited an infrared spectrum typical for silica-gel (Figure 5a). More specifically, siloxane bridge stretching vibration gives a high band at 1100 cm^–1^ and a smaller band at 800 cm^–1^, whereas the band at 950 cm^–1^ is attributed to non-bridging Si–O stretching. The wide band centered around 3500 cm^–1^ is assigned to OH stretching for surface silanol groups and adsorbed water.^33^ Moreover, Si–O–Si bending corresponds to a band at 470 cm^–1.^^70^ BG immobilization noticeably alters the FTIR spectrum of silica nanoparticles (Figure 5b). The presence of the protein is confirmed by amide I and amide II bands, which appear in the 1450–1750 cm^–1^wavelength region. The former, produced by stretching vibration of carbonyl groups of peptide bonds,^71,72^ is slightly displaced with respect to its normal position (1650 cm^–1^ )^42^ due to the overlap with the O–H bending vibration band of adsorbed water (1640 cm^–1^). The latter, centered around 1540 cm^–1^, is due to the N–H in-plane bending and C–N stretching vibrations.^71,72^ As for the spectrum of the SEQ-BG/cell sample (Figure 5c), it showed a remarkable increase in intensity of both amide I and amide II bands, suggesting that the overall amount of enzyme loaded into the silica skeleton has noticeably increased after cellulase adsorption. The amide I band in this spectrum is centered at 1652 cm^–1^, as it is less affected by the influence of the OH band of the adsorbed water. This wavenumber position is the same as that of free cellulase,^41^ indicating a preserved conformation of the adsorbed cellulase with respect to its native form. The most likely mechanism of physical immobilization is the occurrence of hydrogen bonds between the enzymes and the silica support, as already reported for both BG^42^ alone and cellulase.^41^ For BG, electrostatic interaction plays a role since the isoelectric point (pI) of β-glucosidase is around 5.5 while silica has a pI around 3.^73^ For cellulose, the situation is less straightforward since each individual enzyme composing the enzyme complex has its individual isoelectric point, so that some of them are positively charged and others are negatively charged at pH 5.^74^

Enzyme loading was assessed through TGA for both SEQ-BG and SEQ-BG/cell, as reported in Table 2. The SEQ-BG sample reached an enzyme loading of 15 mg/g of support. At the end of the process, the total enzyme loading rose up to 90 mg/g of support, corresponding to an overall 15% YI. Therefore, the finally obtained BG/cellulase weight ratio was equal to 0.2, lower than the desired value of 0.33. The one-pot co-immobilized SIM-BG/cell sample was submitted to TGA as well, with the aim of monitoring any changes in the overall enzyme loading. The result was 100 mg/g of support, corresponding to 20% YI. Considering that the results for SEQ-BG/cell was 90 mg/g and that 15 mg/g is BG and 75 mg/g is cellulase, if we suppose that all the extra uptake is BG, the ratio BG/cellulose would be 0.33, whereas if we consider that all the extra uptake is cellulase, the ratio would be 0.18. We conclude that we cannot precisely know the BG/cellulase ratio in this sample, but we can assume that it is between 0.2 and 0.33. Finally, the BG/cellulase ratio of the SEP-BG/cell sample could be precisely set to 0.33 by mixing the adequate ratio of the two filled vectors.

**Table 2:** Enzyme Loading (mg/g of Support) and BG/Cellulase (w/w) for All the Biocatalysts

biocatalyst	enzyme concentration (mg/mL)	enzyme loading (mg/g of support)	BG/cell (w/w)
SEQ-BG	0.33	15	
SEQ-BG/cell	BG: 0.33	90	0.2
	cell: 1		
SIM-BG/cell	BG: 0.17	100	0.2–0.33
	cell: 1		
SEP-BG/cell	BG: 1	BG/WSN: 150	0.33
	cell: 1	cell/WSN-p: 75	

Results obtained from N_2_ adsorption/desorption experiments point out a progressive filling of the pore structure during sequential immobilization. More specifically, BET surface areas lowered from 544 to 503 to 426 m^2^/g, when evaluated for WSN-p, SEQ-BG, and SEQ-BG/cell, respectively. Similarly, total pore volumes decreased from 1.49 to 1.23 to 1.17 cc/g.

### 3.4 Pre-treated Biomass Hydrolysis

**Figure 6:**
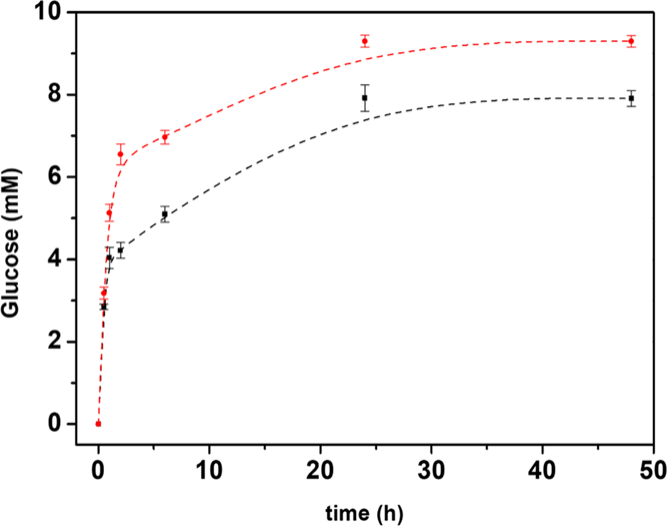
Glucose production over time for free BG:cellulase 1:3 (w/w) (red) and SIM-BG/cell (black). Each experiment was performed in triplicate.

The hydrolysis of the pre-treated biomass is a heterogeneous reaction since the substrate is insoluble while the enzymes are dissolved or suspended in the reaction medium, depending on wether they are in their free or supported form. However, CBH and EG are very prompt in depolymerizing cellulose chains,^75^ leading to a complete disappearance of the floating substrate in about 1 h into the reaction medium. Glucose production over time for free the BG/cellulase mixture in a 1:3 weight ratio is reported in Figure 6 and compared to that achieved by using SIM-BG/cell. Free and supported biocatalysts exhibit the same trend, a linear region in the 0–2 h time interval and a plateau upon approaching 24 h. Final values for glucose concentration are 9.3 and 7.9 mM, corresponding to 97 and 82% YR for the free and supported enzyme mixture, respectively. The difference in terms of long-time performance is slightly higher than that evaluated in short times. Indeed, the activity of SIM-BG/cell is 72 μmol/min·g and is 10% lower than that of the free enzyme mixtures, which is 80 μmol/min·g.

**Figure 7:**
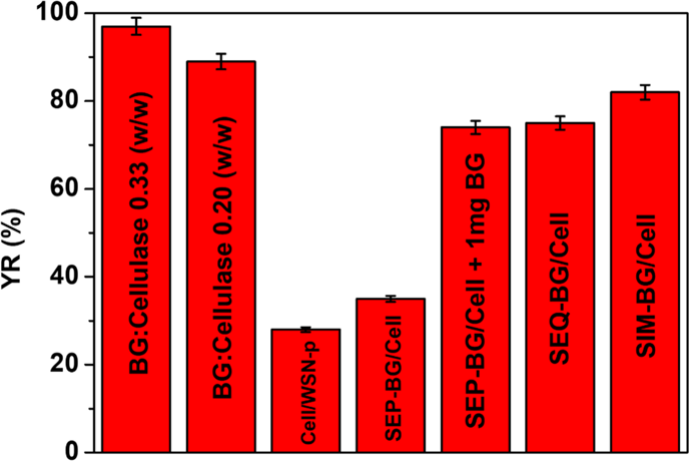
Histograms reporting the yield of reaction obtained by all the selected biocatalysts. Data are shown with error bars. Each experiment was performed in triplicate.

Figure 7 shows histograms representing the glucose yield of each tested biocatalyst. Glucose yield (weight %) is obtained after subtracting from the pre-treated biomass total weight of the ash fraction, determined by TGA. Free cellulase supplemented with BG/cellulase 0.33 and 0.2 w/w shows a glucose yield of 97 and 89%, respectively. Even in this case, as for the CMC, a small conversion increase is confirmed in the 0.33 sample compared to the 0.2 sample, equal to about 9%. BG/cellulase w/w equal to 0.20 was tested in the hydrolysis of untreated biomass. However, the yield of reaction was only 4%, meaning that pre-treatment is necessary to increase cellulose digestibility.

A dramatic drop in conversion yield occurs for cellulase immobilized on WSN-p (cell/WSN-p, 28%) and supplemented with a 0.33 ratio of BG immobilized on WSN (SEP-BG/cell, 35%). The yield rises again to 74% by adding 1 mg of free BG during the reaction catalyzed by SEP-BG/cell. This means that the low yield of these two catalysts depends on a BG deficiency in the enzymatic cocktail, which may be due either to an actual absence of BG inside the support (cell/WSN-p) or to the fact that BG fails to act before the cellobiose accumulates and inhibits the reaction. Cellulase from *T. reesei* contains at least two CBH, five EG, and one BG.^17^ Each of them has its own tertiary structure and physical–chemical properties. Adsorption from an enzyme mixture is a rather complicated process. The final surface composition of the support will depend on the molecular weight and shape of the enzymes, their concentration (that accounts for diffusion from the bulk solution to the support surface), and their different affinity with the surface (i.e., different isoelectric points of each enzyme^38^). Although the molecular weight of BG is comparable with that of other cellulase enzymes, its concentration is significantly lower (1% wt of the mixture^17^). It is therefore likely that during the immobilization of the cellulase on the WSN, BG is partially excluded from the process and the enzymatic cocktail obtained on the surface of the support ends up with a serious deficiency of BG activity. This would explain the low glucose yield reached by using cell/WSN-p. On the other hand, the addition of exogenous BG immobilized on a separate vector (SEP-BG/cell) only slightly improves the yield since in this case, the BG fails to perform its synergistic action. In fact, with individually immobilized enzymes, the cellobiose produced by CBH must diffuse from the pores of one vector to those of the other vector, becoming diluted in bulk solution.^76^ Meanwhile, in the pores of the support, cellobiose is produced at high rate, and its concentration can be enough to inhibit CBH activity. By adding free BG, the problem is mitigated since it can diffuse freely where cellobiose is being produced.

With the co-immobilized and sequentially immobilized systems, the glucose yield rises to 82 and 72%, respectively. Again, we found a difference of about 10% between the two biocatalysts that made us suppose that it could depend on a different BG/cellulase ratio, as reported in Table 2. In both cases, the glucose yield is about 15% lower than the respective free references (free BG/cellulase = 0.2 for SEQ-BG/cell and free BG/cellulase = 0.33 for SIM-BG/cell). This may be due to diffusional limitation or pore blocking, because part of the enzyme complex is located deep inside the pores and cannot be reached by the substrate. In fact, as we will see, the co-immobilized enzyme is very stable. Thus, we tend to exclude that the decrease in yield may be due to deactivation caused by conformational changes of the polypeptide chains. Moreover, SIM-BG/cell and SEQ-BG/cell exerted comparable activities and about only 10% lower than those of the free enzyme mixtures, equal to 72 and 70 μmol/min·g, respectively. The activity of the immobilized biocatalysts scaled in a similar way to conversion with respect to the free enzymes. The results obtained are in agreement with previously available literature results for similar systems. Wang et al. designed a sequential co-immobilization system able to push filter paper conversion to glucose from 40% to 71% in 48 h when integrating cellulase with BG with a BG/cellulase ratio w/w equal to 0.5.^21^ Carli et al. covalently immobilized BG and EG either separately and simultaneously on ferromagnetic nanoparticles, finding a 1.6 fold degree of synergism against pre-treated sugarcane bagasse.^77^ Song et al. covalently immobilized BG and CBH on superparamagnetic nanoparticles finding a retention of activity equal to 67.1 and 41.5% of the free enzymes, respectively.^78^ The advantage of our work lies in the higher yields compared to those reported in the literature as well as in the straightforwardness of the process, which uses a simple physical adsorption on easily synthesized nanoparticles. The higher yields obtained in this work may depend on different features of the system: (i) physical adsorption preserves the native conformation of the enzyme, and (ii) wrinkled nanoparticles favor diffusion of the cellulose chains rapidly depolymerized by CBH and EG that have been adsorbed in proximity of the pore openings. Finally, the results obtained confirm the synergistic action of BG and cellulase. When the enzymes are intimately mixed, as in SEQ-BG/cell and SIM-BG/cell, BG can immediately hydrolyze the cellobiose produced, preventing its concentration from rising to levels that become inhibitory for CBH.

### 3.5 Operational and Thermal Stability

**Figure 8:**
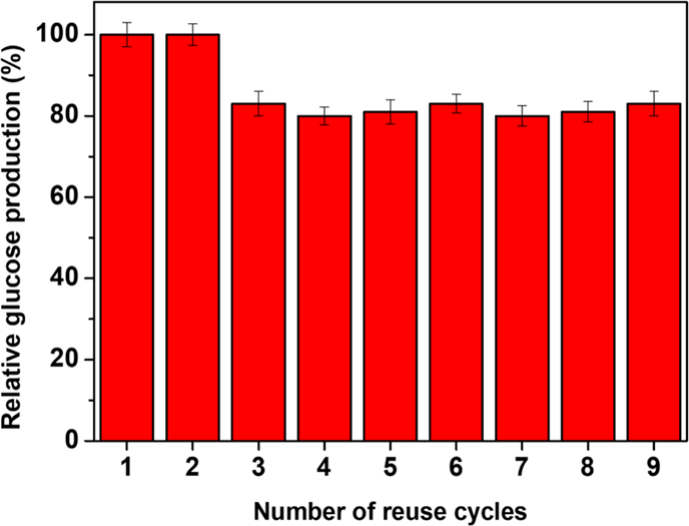
Histograms showing the relative glucose production after each reaction cycle. Each experiment was performed in triplicate.

Operational stability measurements are necessary to assess the reusability of the supported protein in consecutive reaction cycles. The possibility to recover the enzyme from the reaction medium and use it repetitively can balance the high costs associated with the production of the biocatalyst. Figure 8 reports the relative glucose production histograms for the supported one-pot co-immobilized enzymes (SIM-BG/cell).

Results highlight the excellent reusability exerted by the supported biocatalyst, which was reused in 9 consecutive reaction cycles, experiencing only 17% loss in glucose yield. The loss occurs after the second cycle and remains constant up to the 9th cycle. The possible reason for this behavior can be attributed to the mechanism of physical immobilization onto the surface of nanosilica structure. The first enzyme molecules loaded in the adsorption medium diffuse inward into the hierarchical pore structure and gradually fill the inner core of the nanostructure by interaction with the silica surface.^38,41^ When the pores are filled, exceeding enzyme establishes intramolecular aggregates, which do not interact with the inorganic surface. These aggregates are loosely bound to the outer adsorbed enzyme layer and might be easily leached during the reaction,^79^ leading to a slight decrease in the catalytic performances in consecutive reuses. The high stability of the co-immobilized enzyme is surprising if compared to what was previously found for single-enzyme immobilization: BG adsorbed into WSNs exhibited 40% retention of activity after the 5th reuse cycle,^80^ whereas cellulase exhibited a small but gradual loss after the 4th reuse.^41^ Several authors reported the remarkable operational stability of the co-immobilized enzyme systems. BG and cellulase co-immobilized onto hierarchical polymeric microparticles exhibited 75% retention of the original activity in the hydrolysis of CMC after 10 recycles and 57% retention in the hydrolysis of filter paper after 5 recycles.^21^ Co-immobilization of BG and EG resulted in a biocatalyst retaining a relative activity of 80% after the 5th cycle, 2-fold and 8-fold that achieved by single immobilized EG and BG, respectively.^77^ The supported multienzyme system introduced in this work exhibits similar or even better reusability performances if compared to the ones cited above but without using any covalent interaction between protein and support. This proves the effectiveness of physical co-immobilization into WSN-p in producing a high-performance and reusable biocatalyst. Moreover, the reuse allowed preserving almost all the original enzyme loading as confirmed by TGA measurements, indicating that the leaching falls below the sensitivity threshold of the instrument (in the order of 1 μg).

**Figure 9:**
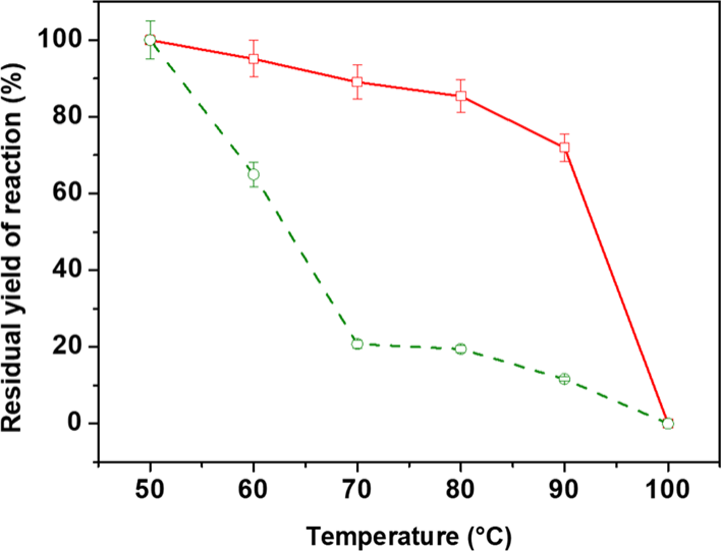
Residual yield of reaction over temperature for the supported (red, solid) and free (green, dashed) biocatalyst. Each experiment was performed in triplicate.

Thermal stability is also an important feature for industrial use of biocatalysts, where they could be exposed to harsh temperatures. Thermal stability is often improved by protein–support interaction. Figure 9 reports the comparison between the thermal stability profiles of the supported and free enzyme mixture.

Free enzymes experience a rapid decrease of residual yield of reaction in the 50–70 °C temperature range, suggesting irreversible modification in protein conformation. For temperatures higher than 70 °C, the activity keeps lowering with a slower rate to the 0% value, recorded at 100 °C. As for supported enzymes, they are remarkably less sensitive to temperature variations as the worsening of the catalytic performances is definitely contained. Indeed, 89% retention of the yield of reaction is recorded at 70 °C, whereas free proteins are almost completely deactivated at the same temperature. Moreover, a 28% total loss occurs for temperatures as high as 90 °C. However, the rising of the temperature up to 100 °C led to a collapse of the catalytic activity, maybe due to on–off denaturation phenomena. An improvement of enzyme thermal stability upon immobilization has been often observed.^81,82^ The interaction of the enzyme with the support can rigidify the enzyme structure by inhibiting the conformational freedom and thermal vibration of the polypeptide chain. In case of enzyme entrapped in a porous support, the interaction with the pore walls further increases enzyme rigidity.^83^ 50 °C is confirmed as the optimal temperature for the dual enzyme system.^21^ Moreover, the confinement into the silica skeleton is proven to be a proper strategy to preserve the structural pattern of cellulolytic enzymes from thermal denaturation.^43,46^

## 4 Conclusions

In this paper, a simple and efficient strategy to co-immobilize BG and cellulase for enhanced conversion of cellulose to glucose was designed. The BG/cellulase ratio was optimized: it was found that a ratio between 0.20 and 0.33 w/w was enough to promote efficient hydrolysis. The two enzymatic systems were immobilized separately or co-immobilized. The synergistic action of BG and cellulase was maximized when the enzymes were intimately mixed, as obtained in SEQ-BG/cell and SIM-BG/cell. In these biocatalysts, BG could hydrolyze cellobiose as soon as it was produced, relieving CBH inhibition. Cellulose hydrolysis yields obtained for SEQ-BG/cell and SIM-BG/cell were 72% and 85%, respectively. The biocatalysts showed a very good operational stability, preserving 83% of the initial yield of reaction for up to nine reuses and better stability in a wide range of temperatures than free enzymes, preserving 72% of the initial yield of reaction at temperatures up to 90 °C. This proves the effectiveness of physical co-immobilization of BG and cellulase into WSN-p for industrial application in biorefineries.
